# Thrombolytic effects of *Douchi* Fibrinolytic enzyme from *Bacillus subtilis* LD-8547 in vitro and in vivo

**DOI:** 10.1186/1472-6750-12-36

**Published:** 2012-07-02

**Authors:** Jun Yuan, Jing Yang, Zhenhong Zhuang, Yanling Yang, Ling Lin, Shihua Wang

**Affiliations:** 1 Key Laboratory of Biopesticide and Chemical Biology, Ministry of Education, Fujian Agriculture and Forestry University, Fuzhou, 350002, China; 2 College of Life Sciences, Fujian Agriculture and Forestry University, Fuzhou, 350002, China

**Keywords:** Thrombolytic effects, *Douchi* Fibrinolytic enzyme, in vitro, in vivo

## Abstract

**Background:**

Today, thrombosis is one of the most widely occurring diseases in modern life. Drugs with thrombolytic functions are the most effective methods in the treatment of thrombosis. Among them, *Douchi* fibrinolytic enzyme (DFE) is a promising agent. DFE was isolated from *Douchi*, a typical and popular soybean-fermented food in China, and it can dissolve fibrin directly and efficiently. A strain, *Bacillus subtilis* LD-8547 produced DFE with high fibrinolytic activity has been isolated in our lab previously.

**Results:**

In the study, thrombolytic effect of DFE from *Bacillus subtilis* LD-8547 was studied in vitro and in vivo systematically. The results showed that DFE played a significant role in thrombolysis and anticoagulation in vitro. And the thrombolytic effects correlated with DFE in a dose-dependent manner. In vivo, the acute toxicity assay showed that DFE had no obvious acute toxicity to mice. Test of carrageenan-induced thrombosis in mice indicated that the DFE significantly prevented tail thrombosis, and arterial thrombosis model test indicated that *Douchi* fibrinolytic enzyme DFE had thrombolytic effect on carotid thrombosis of rabbits in vivo. Other results in vivo indicated that DFE could increase bleeding and clotting time obviously.

**Conclusions:**

The DFE isolated from *Bacillus subtilis* LD-8547 has obvious thrombolytic effects in vitro and in vivo. This function demonstrates that this enzyme can be a useful tool for preventing and treating clinical thrombus.

## Background

Nowadays, cardiovascular diseases including hypertension, coronary heart disease, atherosclerosis and acute myocardial infarction are the leading causes of the human death in the world. Among all kinds of cardiovascular diseases, thrombosis is a frequently occurred symptom [1]. For thrombolytic therapy, the main drugs are urokinase (UK), streptokinase (SK) and tissue plasminogen activator (t-PA). But they are too expensive and their half-life is short. Besides, they are just fit for injection and have the side effect of hemorrhage. In 1897, Nattokinase was extracted from natto [2], and it can be absorbed by oral and injection. From then on, much more attention was paid to the development of new types of fibrinolytic enzyme such as enzymes isolated from Tofuyo [3], Korean Chung kook-jang soy sauce [4], edible honey mushroom [5], Canada blancan [6] and Chinese *Douchi*[7].

*Douchi* fibrinolytic enzyme (DFE) is isolated from *Douchi*, a typical and popular soybean-fermented food in China, and it can dissolve fibrin directly and efficiently. At the same time, DFE can activate t-PA in vivo. Furthermore, it has no toxic and other side-effects, and will not induce hemorrhage in vivo. The molecular weight of DFE is low, so it not only can be absorbed directly in alimentary canal, but also can be used by oral. Therefore, the study and development of DFE have significance for treating clinical thrombus [8,9].

A strain, *Bacillus subtilis* LD-8547 produced DFE with high fibrinolytic activity has been isolated in our lab previously [10,11]. In this paper, the thrombolytic effects of this DFE in vitro and in vivo were studied respectively.

## Methods

### Medium

The agar slant medium consisted of (w/w): beef extract 0.5%, peptone l%, NaC1 0.5%, agar 1.5%, pH 7.0. The slant was incubated at 37°C for 24 h. The flask culture medium contained (w/w): rice power 5%, soybean power 4%, NH_4_NO_3_ 0.5%, CaC1_2_ 0.01%, MgSO_4_ 0.7%, K_2_HPO_4_ 0.4%, KH_2_PO_4_ 0.2%, pH 7.0 [4,12].

### Purification of DFE

*Bacillus subtilis* LD-8547 was grown at 32°C in a 100 mL flask containing 50 mL culture medium at 150 r/min for 72 h. DFE was purified by a previously described method of Wang et al. [13] with a series of procedure including salt-out, dialysis and gel filtration chromatography with Sephadex G-100.

### Fibrinolytic activity assay

Amidolytic activity of the DFE was estimated using synthetic substrate (Suc-Ala-Ala-Pro-Phe-pNA) [7]. The reaction mixture containing 8 μL of 1 mM synthetic substrate, 40 μL of 20 mM Tris–HCl buffer (pH 8.0) and 6 μL of enzyme solution was incubated at 37°C for 10 min. The absorbance of released pNA at 405 nm was measured with a microplater. One unit of the amidolytic activity was defined as the amount of enzyme that liberated 1 ng of p-nitroanilide per minute. Protein was determined using Folin Ciocalteau phenol reagent.

### Thrombolytic effects of DFE in vitro

#### Anticoagulant effect of DFE on animal blood

Fresh animal blood samples (rat, rabbit and sheep) were collected, and different doses of DFE (2070 U, 1035 U, 518 U and 259 U) were added into these samples, respectively. The control was added with 1 mL Tris–HCl. After mixed gently, the mixture was incubated at 37°C for 30 min. Then the anticoagulant effects of DFE on animal blood were observed.

#### Clot lytic effect of DFE

Clot lytic effect of DFE was studied with natural clot in vitro. The animal blood clot was cut into the same size, and 5000 U urokinase (as a positive control), Tris–HCl buffer (as a negative control) and different doses (414 U, 621 U, 1035 U and 2070 U) of DFE were added. The mixture was incubated at 37°C for 24 h. Then aliquots were taken from the reaction mixture for analysis [14].

#### Euglobulin lysis experiment

According to Cheng and Buckell [15,16], 0.5 mL plasma was added into in a centrifuge tube containing 9 mL distilled water. The pH was adjusted to 4.5 by adding 0.1 mL of 1% acetic acid. The mixture was placed at 4°C for 10 min. After euglobulin fraction of the plasma was precipitated, the tubes were centrifuged at 3000 r/min for 5 min. The supernatant was decanted, and 0.5 mL borate solution (pH 9.0) was added to the precipitate. The mixture was placed at 37°C for 2 min and stirred gently with a glass rod. Also, 0.5 mL of 0.025 M calcium chloride solution was added to form euglobulin clots. Then DFE was added to the clots at different doses (469 U, 938 U and 1877 U). The tubes were incubated at 37°C, and the lysis of clots was inspected after 2.5 h.

#### The hemolysis test of blood erythrocytes

Red blood cell suspension (2%) was prepared according to Chinese Pharmacopoeia (2010 edition) [17]. And different doses (207 U, 414 U, 621 U, 828 U and 1035 U) of DFE were added into it respectively. Normal saline (NS) and distilled water were added as controls. After mixed gently, the mixture was incubated at 37°C. After 6 h, the absorbance of supernatant was determined at 545 nm by spectrophotometer with distilled water as a blank control. Hemolysis effect was determined by the formula as following:

(1) $$\text{hemolysis rate}\left(\%\right)=\left(\text{ODt}–\text{ODnc}\right)/\left(\text{ODpc}–\text{ODnc}\right)\times 100\%.$$

ODt was tested absorbance. ODnc was negative absorbance. ODpc was positive absorbance. If hemolysis rate exceeds 5%, it demonstrates that the DFE dose is not suitable for injection.

### Thrombolytic effects of DFE in vivo

#### Acute toxicity assay

Kunming mice and New Zealand rabbits used for animal experiment were purchased from the Shanghai Laboratory Animal Center, and all animal work was performed according to relevant national and international guidelines. All animal experiments were approved by the Animal Ethics Committee of the Fujian Agriculture and Forestry University. In order to evaluate the acute toxicity of DFE on adult mice,tests were carried out according to the method described by Wang [11]. Twenty female and twenty male mice (20 ± 2 g) were housed in stainless steel cages in a ventilated animal room. Distilled water and sterilized food for mice were available ad libitum.

They were acclimated to this environment for 7 days prior to dosing. Mice were randomly divided into four groups: control groups (female and male) and experimental groups (female and male). Before treatment, mice were fasted overnight. Subsequently, the control groups and the experimental groups were given NS and DFE respectively by mouth, and then were provided with food and water 2 h later. The symptom and mortality were observed and recorded carefully in 2 weeks.

#### Thrombolytic effect of DFE on mouse thrombosis model

Male Kunming mice were randomly divided into 4 groups. Group 1 served as control was treated with NS. Groups 2, 3, 4 were given 2051, 4103 and 8206 U/35 g bw (body weight) purified DFE dissolved in NS by oral administration for a week, respectively. Half an hour after the last treatment with the DFE, 17 μL/10 g bw carrageenan were injected through celiac. The thrombus lengths were measured at 24 h. Each experiment was done at a minimum in triplicate [18].

#### Effect of DFE on bleeding and clotting time

Mice were divided into four groups randomly (five mice were used per group) and treated with NS and different doses of DFE (2206 U, 4412 U and 8824 U) by mouth for a week respectively. The bleeding time was measured by using a standard incision in the tail of mice. Mice were fixed, and then 3 mm tail tips were cut. When the tail was bleeding, the blood was wiped every 30 s. The bleeding time was measured until bleeding stopped naturally.

The clotting time was determined using the method previously described [19]. An hour after the last treatment with different doses of DFE, a drop of blood was put on the clean slide. Then the blood was stirred by dry pinhead every 30 s. The clotting time was measured until fibrin filament was stir out.

#### Lytic effect of DFE on whole blood clot and plasma clot

Mice were divided into four groups randomly (five mice were used per group) and treated with NS and different doses of DFE (4100,8200,16400 U/35 g bw) by oral for a week respectively. Half an hour after the last treatment, 1 mL blood was collected from eyes. The blood was incubated at 37°C until it was clotted. Then the lysis of blood clots was observed after 4 h.

Plasma clots were prepared as followed: the blood (1 mL) were added with sodium citrate (0.2 mL, 3.8%) and then centrifuged at 3000 r/min for 10 min. Plasma was separated (0.4 mL) and incubated at 37°C adding with 8 U of thrombin to form clot. The lytic effect of DFE on plasma clots was observed after 3 h.

#### Effect of DFE on carotid artery thrombosis

Ten New Zealand rabbits were divided into two groups: control group and DFE group. DFE group were fixed and their carotid arteries were exposed after anesthetizing by 20% urethane (5 mg/kg). And then, thrombosis was formed by covering a piece of filter paper (1.0 cm × 1.5 cm, with 10% FeCl_3_) on the carotid artery for 15 min. At the same time, the rabbits were treated with DFE (1035 U/kg) by abdominal injection. The effect of DFE on the carotid thrombosis was observed at different time [20].

## Results

### Purification of DFE and fibrinolytic activity assay

**Figure 1:**
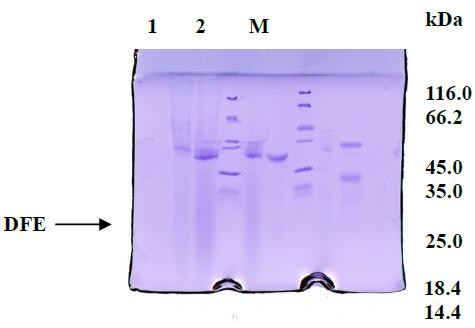
**SDS-PAGE assay of DFE protein.** M: Protein Marker; Lane 1: DFE Unpurified by Sephadex G-100; Lane 2: Purified DFE by Sephadex G-100.

DFE was purified from *Bacillus subtilis* LD-8547 with a series of procedures. The activity of DFE was estimated with synthetic substrate. And the results showed that the fibrinolytic activity of DFE reached 21750 U/mL after condensed (Figure 1).

### Thrombolytic effects of DFE in vitro

#### Anticoagulant effect of DFE on animal blood

**Figure 2:**
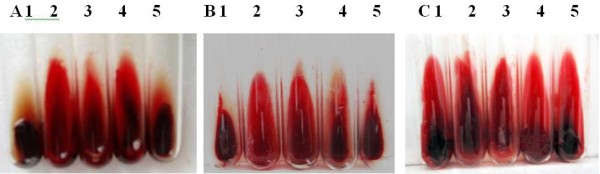
**Anticoagulant effect of DFE with different concentrations on animal bloods in vitro. A-C** was rabbit blood, rat blood and sheep blood, respectively. 1: added with 1 mL Tris–HCl ; 2–5: added with 2070 U, 1035 U, 518 U and 259 U of DFE respectively.

The anticoagulant effects of DFE on animal blood were tested. The results indicated that low dose DFE (259 U) had no notable effect on clot of rabbit blood, but anticoagulant effect was gradually enhanced with the increasing dose of DFE (Figure 2A). With 518 U and 1035 U of DFE, the bloods were coagulated partly. And anticoagulant effect was very obvious when DFE concentration reached 2070 U. The blood was fluid and not clotted.

Anticoagulant effects of DFE on other animal blood such as rat and sheep were similar to that of rabbit blood (Figure 2B, 2C).

#### Clot lytic effect of DFE

**Figure 3:**
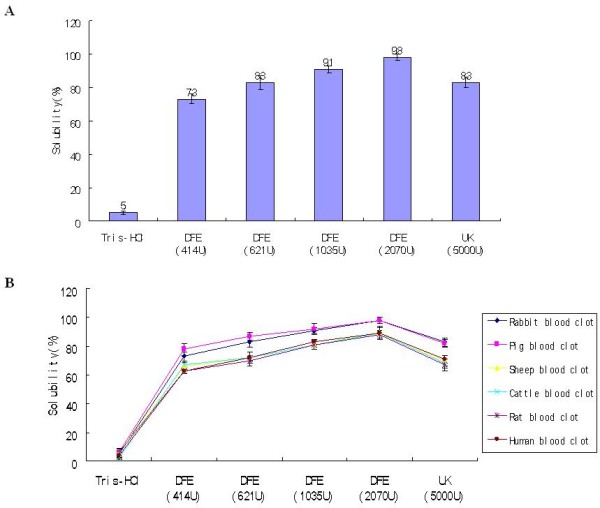
**Thrombolytic effects of different doses of DFE on animal blood clot in vitro. A**: The solubility rate of rabbit’s blood clot after treated with DFE (414 U, 621 U, 1035 U and 2070 U) and UK (5000 U). **B**: The solubility rate of different animal blood clots after treated with DFE (414 U, 621 U, 1035 U and 2070 U) and UK (5000 U). UK: urokinase.

Solubility rate of blood clots were detected by adding different doses of DFE to prepared rabbit blood clots. And the results showed that DFE lysed the clots with solubility rate of 73%, 83%, 91% and 98%, respectively, compared with 83% of UK and 5% of negative control (Figure 3A).

The clot lytic effect on other blood clots of rat, pig, sheep, cattle and human was similar to that of rabbit blood (Figure 3B). These results suggested that DFE had obvious effect on dissolving the blood clot, and the effect was enhanced as the concentration of DFE increased.

#### Euglobulin lysis experiment

**Figure 4:**
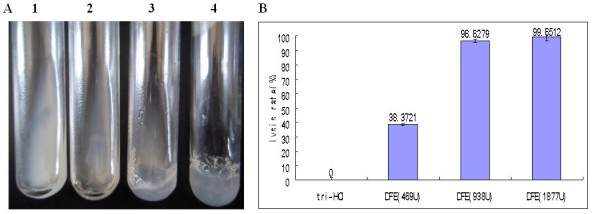
**The euglobulin lysis by DFE. A**: 1–3: Euglobulin added with DFE of 1877 U, 938 U and 469 U respectively; 4: Control group adding with Tris–HCl. **B**: The weight of the rest fibrin clot after treated with Tris–HCl and different dose of DFE (469 U, 938 U and 1877 U).

The lysis effect of DFE on euglobulin was determined by weighing the wet weight of the residual euglobulin clots after treated with DFE. The results showed that low dose DFE (469 U) could dissolve part of the clot (38.4%), and higher dose DFE (938 U and 1877 U) lysed almost all the euglobulin clots (96.6% and 99.3%) after 2.5 h treatment (Figure 4A, 4B).

#### The hemolysis test of blood erythrocytes

**Figure 5:**
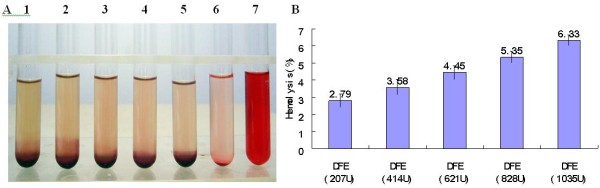
**The hemolysis test on blood erythrocytes of rabbit. A**: Rabbit erythrocyte added with different agent. 1–5: Rabbit erythrocyte added with 207 U, 414 U, 621 U, 828 U and 1035 U of DFE, respectively. 6: NS; 7: Distilled water; **B**: The hemolysis rate of rabbit erythrocytes added with DFE.

Different doses of DFE were added to erythrocytes and then hemolysis rate was tested. The results indicated that there were no obvious hemolysis and agglutination effects on rabbit erythrocytes (Figure 5A). Hemolysis rates of high dose DFE (828 U and 1035 U) were a little more than 5% (5.35% and 6.33%, respectively), but low dose DFE (207 U, 414 U and 621 U) did not hemolyze erythrocytes obviously, and their hemolysis rates were less than 5% (Figure 5B). So they were safe and fit for intravenous injection.

**Figure 6:**
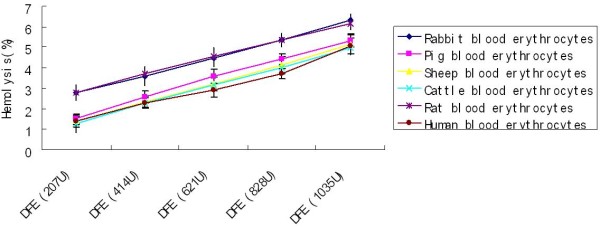
The hemolysis rate of different animal blood erythrocytes after added with different doses of DFE.

The haemolytic effect of DFE on rat, pig, sheep, cattle and human blood erythrocytes was similar to that of rabbit blood on the whole (Figure 6).

### Thrombolytic effects of DFE in vivo

#### Acute toxicity assay

**Figure 7:**
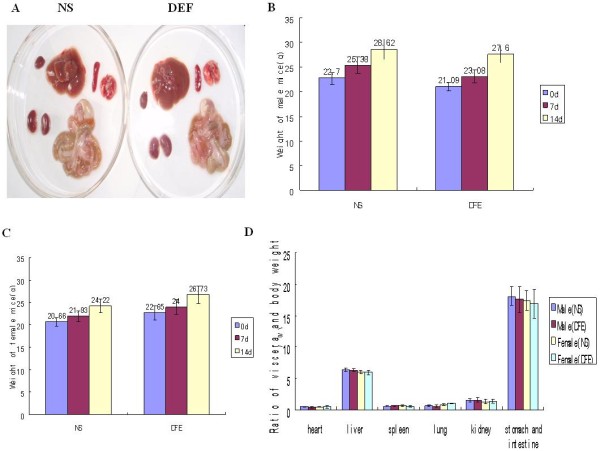
**The acute toxicity test of DFE. A**: the morphology of mice viscera after given DFE for two weeks. **B**: the weight of male mice. **C**: the weight of female mice. **D**: Ratio of viscera to body weight.

The mice were given 691,586 U/Kg DFE for 2 weeks, and then the weight of mice body and various organs were detected. As a result, DFE showed no obvious acute toxicity to mice. Morphology of mice viscera given with DFE were nearly the same to the control, and there were no abnormal changes of the pathologic section in the hearts, livers, spleens, lungs, kidneys, stomachs and intestines of all mice (Figure 7A). Also, no obvious differences were found in the body weight and viscera (P >0.05) (Figure 7B-7D).

#### Thrombolytic effect of DFE on mouse thrombus model

**Figure 8:**
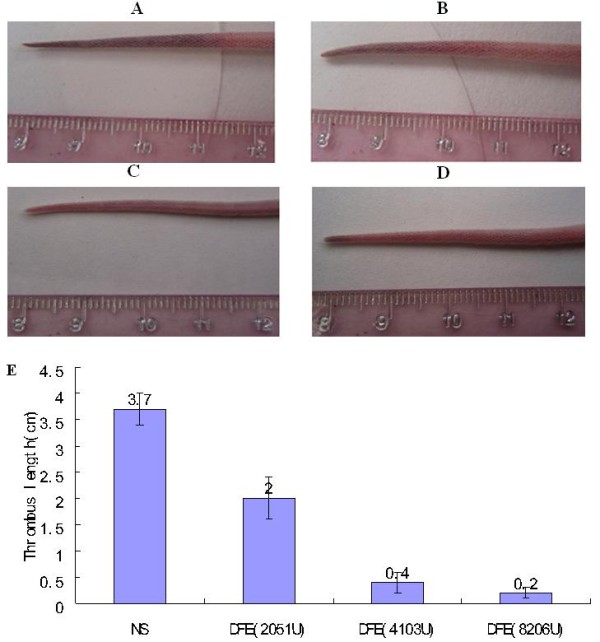
**Thrombolytic effect of DFE on tail thrombus model caused by carrageenan. A**: Tail thrombus model. **B-D**: The morphology of mice tail after given DFE for 24 h (2051 U/35 g bw, 4103 U/35 g bw , 8206 U/35 g bw). **E**: The length of mice tail thrombus after injecting DFE.

In order to detect the thrombolytic effect of DFE in vivo, Carrageenan-induced tail thrombus model was used. Tail thrombus was formed at 24 h after the injection of carrageenan (Figure 8A). After treated with different dose DFE according to Methods, mice were subcutaneously injected with carrageenan, and then length of tail thrombus was measured at 24 h. It was showed that DFE could significantly inhibit thrombus formation in carrageenan-induced thrombosis model in mice (Figure 8B-8D). The average thrombus length in group 1 was 3.7 cm, and the average length of thrombus decreased sharply in groups 2 (2 cm) and 3 (0.4 cm), respectively, with the increasing amount of DFE. The thrombus nearly disappeared in the tip of mouse tail in group 4(Figure 8E). The results suggested that DFE can prevent tail thrombosis induced by carrageenan, and the effect was enhanced with the increasing dose of DFE.

#### Effect of DFE on bleeding and clotting time

**Figure 9:**
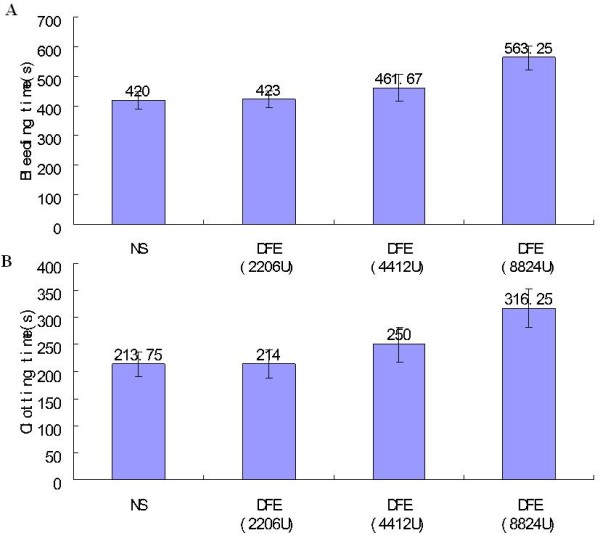
Bleeding time (A) and clotting time (B) of mice treated with DFE (2206U, 4412U and 8824U).

In vivo assay, it showed that the enzyme could affect both the bleeding and clotting time of experimental mice, and there was a positive correlation between the time and the dose of DFE. The bleeding and clotting time were postponed obviously when treated with high dose of DFE (p < 0.05). Also low dose and middle dose of DFE could postpone the time, but had no marked effect compared to the control group (Figure 9A, 9B).

#### Lytic effect of DFE on whole blood clot and plasma clot

**Figure 10:**
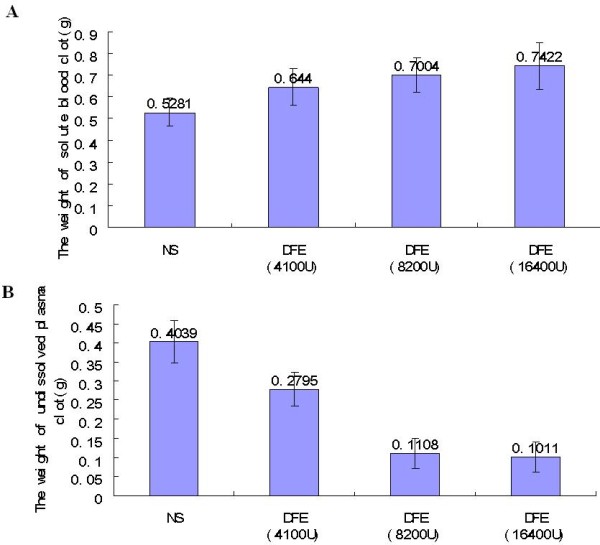
**Lysis of the whole blood clot and plasma clot. A**: the weight of solute blood clot after mice were treated with different dose of DFE (4100 U, 8200 U and 16400 U); **B**: the weights of undissolved plasma clot after mice were treated with different dose of DFE (4100 U, 8200 U and 16400 U).

The results (Figure 10A) showed that the enzyme could lyse whole blood clot as well as plasma clot within 30 min. There was a dose-dependency relationship between DFE and solubility of blood clots. The higher dose (8200 U and 16400 U) of DFE could increase the solubility of blood clots markedly (p < 0.05), but the low dose (4100 U) of DFE had no obvious effect (P > 0.05). Also, lytic activity of DFE to plasma clot became higher as the dose increasing. Compared to the control, plasma clots could be lysed obviously even with low dose of DFE (p < 0.05) (Figure 10B).

#### Arterial thrombosis model

**Figure 11:**
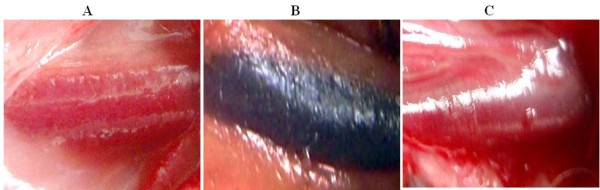
**Arterial thrombosis model test of DFE. A**: Natural carotid arteries. **B**: Arterial thrombosis model made by FeCl_3._**C**: Carotid arteries after thrombosis model were injected with DFE (1035 U/kg) for 2 days.

To assess the thrombolytic effect of DFE in vivo, FeCl_3_ was applied to the exposed carotid artery to elicit an endothelial damage. Before thrombus was made, vessel was bright red and the blood flowed normally (Figure 11A). After the formation of thrombus, vascular wall turned dark obviously, the vessel contracted and blood flowed slowly (Figure 11B). Meanwhile, the results demonstrated that although the thrombus formed vessel wall was a bit darker than normal vessel 2 d after DFE injection, the thrombus formed vessel became bright red and the blood flowed normally again (Figure 11C). It suggested that DFE had favorable thrombolytic effect in vivo.

## Discussion

In recent years, in order to enhance the efficacy and safety of fibrinolytic therapy, many researches were pursued to discover fibrinolytic enzymes from food-grade microorganisms. Among them, much attention was paid to fibrinolytic enzymes isolated from *Douchi.*

In this study, many experiments were carried out to test the thrombolytic effect of *Douchi* fibrinolytic enzyme from *Bacillus subtilis* LD-8547 in vitro and in vivo. The assay demonstrated that in the groups treated with more than 518 U of DFE, the anticoagulation effect was obvious on animal blood, and the effect was enhanced with increasing dose of DFE. The cascade and waterfall hypotheses of blood coagulation indicate that the coagulation process is divided into three stages: the formation of prothrombin activator, thrombin formation and fibrin formation. In this study, the result indicated that DFE can inhibit the extrinsic coagulation system, which may be the key to antithrombotic and thrombolytic activity. And in the low dose group (259 U), there is no or fewer side effects in bleeding and does not affect the body's normal clotting mechanism. Therefore, low dose DFE is recommended for the long-term use to prevent thrombosis.

Antithrombotic or thrombolytic drugs can block the pathway of thrombus formation. The fundamental task of thrombolytic therapy is the degradation of fibrin by plasmin, which can be activated by the activators from inactive plasminogen. SK and UK depend on this indirect activation pathway. In addition to this indirect working mechanism, thrombolytic enzyme can dissolve fibrin directly, which was proved by our thrombolysis experiments in vitro. Our studies demonstrated that DFE displayed strong thrombolytic ability in mouse. Its effect is better than clinically used UK.

Thrombus formed via the effects of cruor, anticoagulation, fibrinolytic system, haemorheology, vascular endothelial cells, platelets and other factors. Animal thrombosis models are the most effective way to evaluate the function of thrombolytic agents. As an experimental model of peripheral obstructive disease, carrageenan-induced thrombosis in mice was used, because of its advantages of simple induction in small laboratory animals and easy to observe without killing the animals. In this study, the results showed that DFE significantly inhibited tail thrombus formation after the injection of carrageenan. The antithrombotic effect of DFE is even more obvious than some newly reported fibrinolytic enzymes such as Subtilisin QK [16].

The oxidative damage of the endarterium induced by FeCl_3_ is the traditional method to establish the Animal Blood Bolts Model, and it is convenient, fast and the results are reproducible. A moderate concentration (10%) of FeCl_3_ was used in this study, and the fibrinolytic system could dissolve fibrin, which is the main component of thrombus. The impacts on the fibrinolytic system are the key to evaluate the effect of thrombolytic drugs. In the study, markedly thrombolytic effect of DFE was showed in the in vivo test after the carotid artery thrombosis was made.

Microorganism is an important source of thrombolytic enzymes. With the fast growth and easy control characteristics, microorganism can be manually controlled to obtain the target product. With the development of fermentation industry and extraction of fermentation products, there will be a broad prospect of clinical use of this thrombolytic agent.

Many factors play important roles in thrombosis. Molecular mechanisms on effective prevention and treatment of thrombosis of the DFE from *Bacillus subtilis* LD-8547 is not clear now. Therefore, a further study is needed in the future.

## Conclusions

The present results extend our previous findings that the DFE from *Bacillus subtilis* LD-8547 has effective fibrinolytic activity on fibrin plates and plasma plates. In addition, in this work, we found that DFE could lyse euglobulin, relieve thrombus symptom on tail of mouse and carotid of rabbit, elongate bleeding and clotting time, and also had an anticoagulant and clot lytic effect on animal blood. Simultaneously, no toxicity to the mice and no perniciasm on erythrocytes were detected. Therefore, taken together the results clearly illustrate that the DFE has promises in clinical applications to prevent and cure the thrombosis and thrombotic related disorders.

## Abbreviations

DFE, Douchi fibrinolytic enzyme; UK, Urokinase; SK, Streptokinase; t-PA, Tissue plasminogen activator; NS, Normal saline.

## Competing interests

The authors declare that they have no competing interests.

## Authors’ contributions

SHW and JY designed the approach, collected data and interpreted the research results. Participated with manuscript preparation and editing. SHW supervised JY, prepared and submitted the manuscript. ZHZ and YLY provided technical assistance with cell culture, DFE preparation and collection. Participated with manuscript preparation and editing. LL provided technical assistance with thrombolytic effect of DFE in vitro. All authors read and approved the final manuscript.
